# Supplementary figures and images for: Quantitative trait loci for grain mineral element accumulation in Vietnamese rice landraces
Source: PLoS One. 2024 Dec 23;19(12):e0315666. doi: 10.1371/journal.pone.0315666 (PMC11666011; doi:10.1371/journal.pone.0315666)

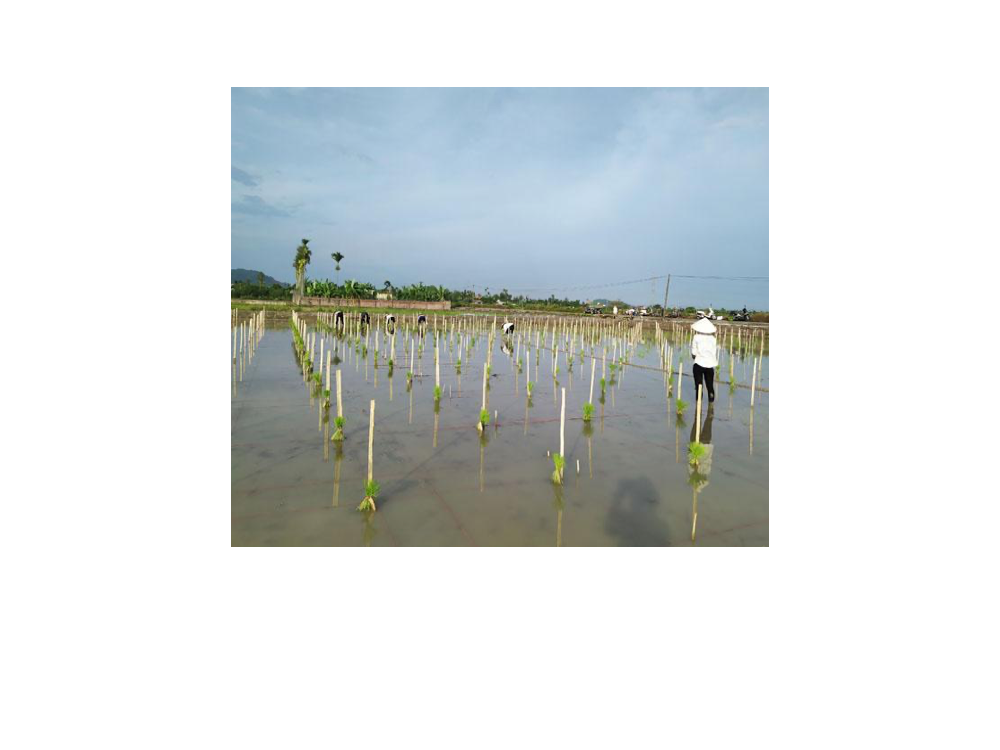

Supplement: S1 Fig — (TIF) [file pone.0315666.s002.tif]

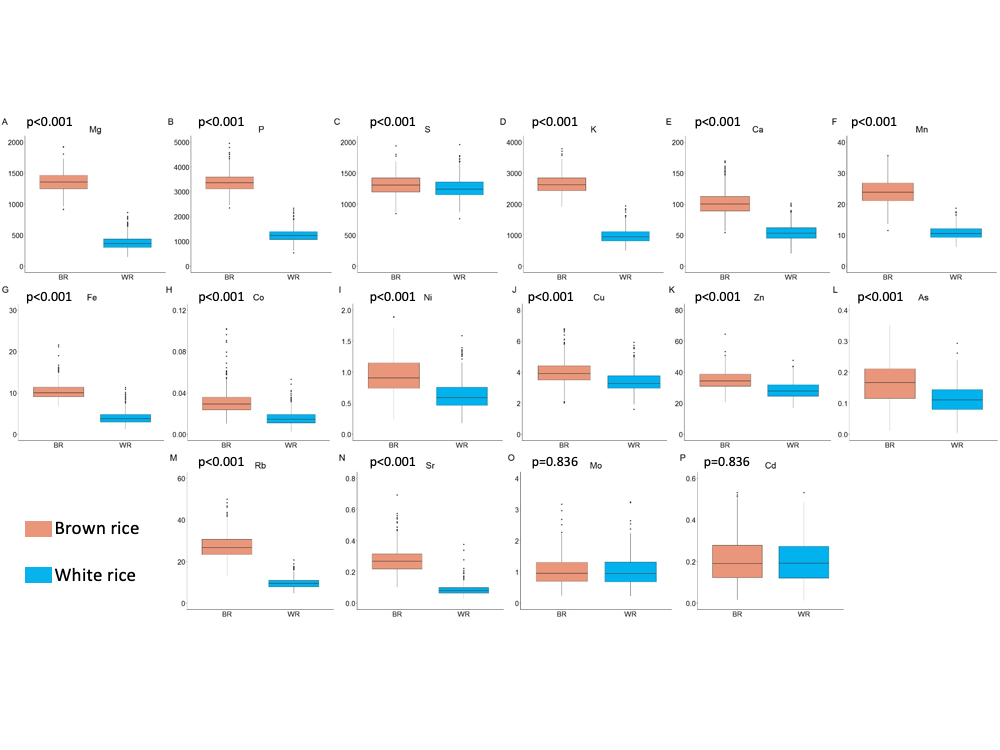

Supplement: S2 Fig — Ion content is represented as ppm. p-values from ANOVA tests are represented. (TIF) [file pone.0315666.s003.tif]

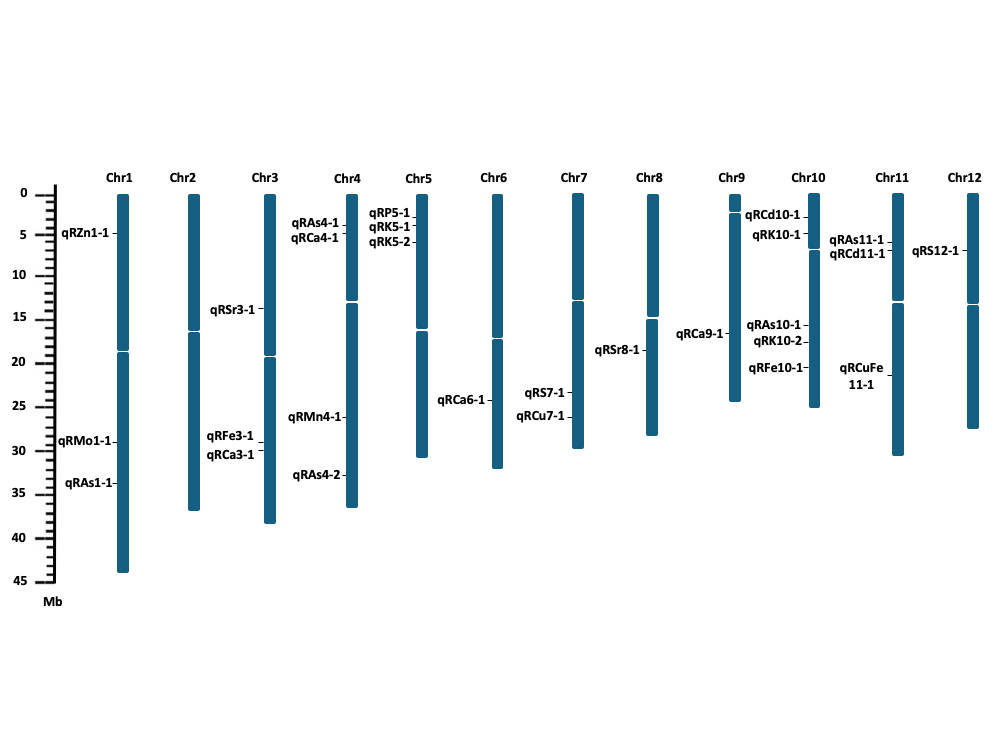

Supplement: S3 Fig — The 12 rice chromosomes are represented. The vertical scale indicates the size of chromosomes in mega base pairs (Mb). (TIF) [file pone.0315666.s004.tif]

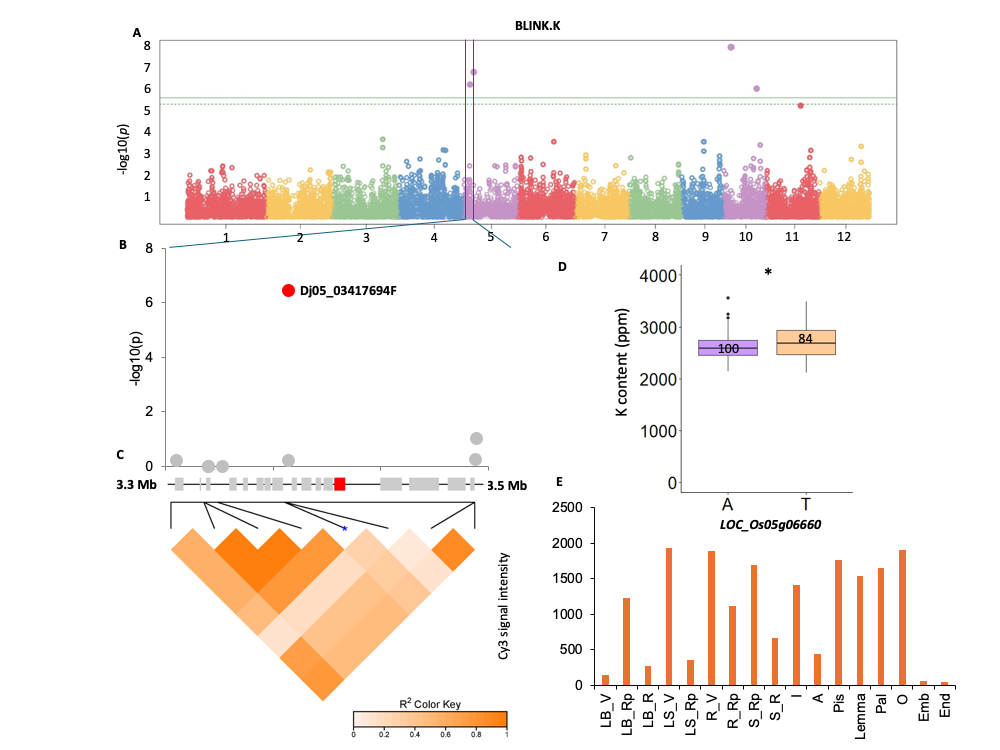

Supplement: S4 Fig — A. Manhattan plot representing the p-values of associations between SNPs and grain K content using the BLINK model in GAPIT. The solid green line represents the Bonferroni threshold and the dotted green line represents FDR threshold. B. Focus on the association between SNP Dj05_03417694F and grain K content on chromosome 5. C. Definition of QTL qRK5-1 based on blocks of linkage disequilibrium around Dj05_03417694F. The genes present in the QTL region are represented by grey rectangles. The selected candidate gene LOC_Os05g06660 is highlighted in red. D. Box plots representing the polymorphism distribution at SNP Dj05_03417694F in the Vietnamese collection. The number of genotypes in each haplotype group is represented. E. Expression levels of LOC_Os05g06660 at different developmental stages. Expression data in different plant tissues were retrieved from the Rice Xpro database [44] as follow; LB_V: Leaf blade at vegetative stage; LB_Rp: Leaf blade at reproductive stage; LB_R: Leaf blade at ripening stage; LS_V: Leaf sheath at vegetative stage; LS_Rp: Leaf sheath at reproductive stage; R_V: Root at vegetative stage; R_Rp: Root at reproductive stage; S_Rp: Stem at reproductive stage; S_R: Stem at ripening stage; I: Inflorescence of 3.0–4.0 mm; A: Anther of 0.7–1.0 mm; Pis: Pistil of 10–14 cm panicle; L: Lemma of 4.0–5.0 mm floret; Pal: Palea of 4.0–5.0 mm floret; O: Ovary 3 days after flowering; Emb: Embryo at 14 days after flowering; End: Endosperm at 14 days after flowering. (TIF) [file pone.0315666.s005.tif]

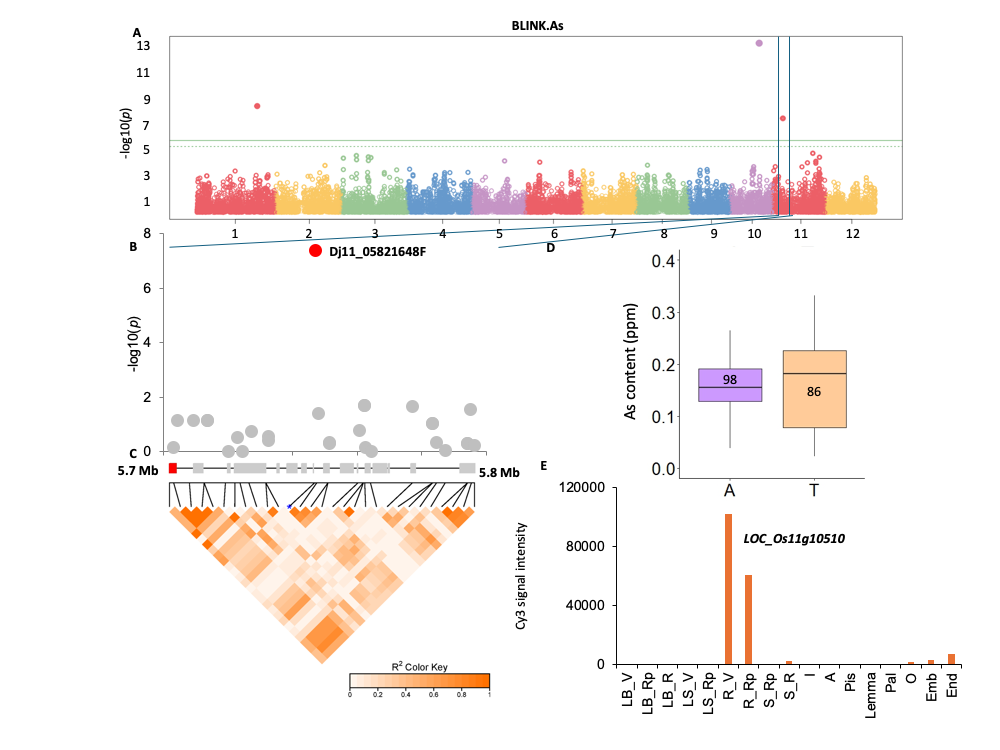

Supplement: S5 Fig — A. Manhattan plot representing the p-values of associations between SNPs and grain As content using the BLINK model in GAPIT. The solid green line represents the Bonferroni threshold and the dotted green line represents FDR threshold. B. Focus on the association between SNP Dj11_05821648F and grain As content on chromosome 11. C. Definition of QTL qRAs11-1 based on blocks of linkage disequilibrium around Dj11_05821648F. The genes present in the QTL region are represented by grey rectangles. The selected candidate gene LOC_Os11g10510 is highlighted in red. D. Box plots representing the polymorphism distribution at SNP Dj11_05821648F in the Vietnamese collection. The number of genotypes in each haplotype group is represented. E. Expression levels of LOC_Os11g10510 at different developmental stages. Expression data in different plant tissues were retrieved from the Rice Xpro database [44] as follow; LB_V: Leaf blade at vegetative stage; LB_Rp: Leaf blade at reproductive stage; LB_R: Leaf blade at ripening stage; LS_V: Leaf sheath at vegetative stage; LS_Rp: Leaf sheath at reproductive stage; R_V: Root at vegetative stage; R_Rp: Root at reproductive stage; S_Rp: Stem at reproductive stage; S_R: Stem at ripening stage; I: Inflorescence of 3.0–4.0 mm; A: Anther of 0.7–1.0 mm; Pis: Pistil of 10–14 cm panicle; L: Lemma of 4.0–5.0 mm floret; Pal: Palea of 4.0–5.0 mm floret; O: Ovary 3 days after flowering; Emb: Embryo at 14 days after flowering; End: Endosperm at 14 days after flowering. (TIF) [file pone.0315666.s006.tif]
