# Supplementary material for: Quantitative trait loci for grain mineral element accumulation in Vietnamese rice landraces
Source: PLoS One. 2024 Dec 23;19(12):e0315666. doi: 10.1371/journal.pone.0315666 (PMC11666011; doi:10.1371/journal.pone.0315666)
Supplement: Attachment — Submitted filename: Authors_Response_Tran_etal_241024.docx [file pone.0315666.s009.docx]

**Reviewer #1:** Quantitative trait loci for grain mineral element accumulation in Vietnamese rice landraces.

Author’s response: We thank the Reviewer for the constructive comments on the manuscript.

\# Title: Rewriting suggested.
Abstract: OK
Keywords: OK

A dedicated Abbreviation section suggested (including transporters, QTLs etc.).
Author’s response: We have added an Abbreviation section and tried to reduce the abbreviations within the text. Gene/protein abbreviations are consistently preceded by their full name. Abbreviations for these were not included because they do not appear throughout the text but rather in specific sections.

Authors are advised to add a note on deprivation of elements among the population (age and sex wise etc.).

Author’s response: We now refer in the first paragraph to Fe and Zn deprivation repartition in the world and population.

Role of As and Cd in minimizing elemental content in raw and cooked rice.
Author’s response: We thank the Reviewer for this comment. We have now added a description of these aspects in the first paragraph of the introduction.

Clearly mention the gap area and aim and scope of the study (bullet).
Author’s response: We have clarified the gap, aim and scope of the study according to the Reviewer’s comment.

Line 133: 50 cm spacing or 5.0 cm spacing?
Author’s response: In the field experiment, we observed 25 cm spacing between and within rows, 50 cm spacing between blocks and 75 cm spacing between flowering groups. We have now clarified that in the manuscript.

SRM recovery should be presented in tabular format in supplementary file.
Author’s response: The ionomic analysis did not include measurements on certified reference material. However, calibration standards from purchased certified standards were used to calibrate the instrument and “pooled sample” (a small amount taken from each of the samples analysed and combined) were analysed after every nine samples in all of the batches of analysis to ensure consistency between analysis batches as indicated in the material and methods. We acknowledge that certified reference material would have been useful to confirm absolute concentration values in our samples but our analysis rather ensured that every sample was treated similarly to allow comparison between genotypes from the panel. Furthermore, we observed values within the order of magnitude of the ion contents usually observed in rice. Therefore, we do not expect major bias in the absolute values of ion content in rice grains we measured in our analysis.

Line:168: mean of how many replications?
Author’s response: It is the mean of three replications. We have added this information in the text.

In the discussion section, authors are required to interpret the varieties having greater elemental profile linked with QTL in terms of RDA or RDI for greater acceptability.
Moreover, how climatic fluctuations can influence the elemental profile will be highly appreciated.

Author’s response: We have mentioned the international breeding targets set for rice Fe and Zn content in the discussion and we added a sentence on the importance of developing climate resilient rice varieties while ensuring grain quality.

**Reviewer #2:** The manuscript entitled “Quantitative trait loci for grain mineral element accumulation in Vietnamese rice landraces” focuses on identifying the QTL regions and candidate genes for different mineral elements in rice landraces. The manuscript is clear, concise, and informative. The methods are appropriate and the results are presented in a logical and transparent manner. The discussion is balanced and comprehensive.

Author’s response: We thank the Reviewer for the constructive comments on the manuscript.

I have only a few minor comments and suggestions for improvement:
Introduction: The Introduction has been well drafted, with extensive collection of points related to the study, highlighting the rationale behind it. However, the chronology of these sentences may have to be organised for clarity. The following corrections may be carried out for improvement of the manuscript,
Comment 1: Line 42–62, 1st paragraph contains the brief introduction about biofortification and its improvement, while 2nd paragraph (line 64-81) contains other elements and its correlation with harmful heavy metals, followed by its improvement. This could be modified as stating brief introduction of different elements in 1st paragraph and improvement strategies in 2nd paragraph.
Author’s response: We have re-organised the two paragraphs according to the Reviewer’s comment.

Comment 2: Line 83, introductory sentences need to be added to this paragraph. Instead of starting with different GWAS studies, mention about other mapping strategies and how advantageous GWAS is over them with respect to grain ions trait.
Author’s response: We added a sentence in the introduction describing the advantages of GWAS over multiparental populations in detecting QTLs associated with ion accumulation.

Comment 3: Line 108, the concluding statement, which defines the objective of the study needs clarity. Just mention the objective and delete the findings, which may be discussed in results section.
Author’s response: We have clarified the objectives and removed the findings.

Materials and methods: The methodology has been presented well in different subsections viz., Plant materials and genotypic data, Field experiment, Grain ion phenotyping, Statistical analysis, Genome-wide association studies and Linkage disequilibrium (LD) heatmap and identification of candidate genes. However, the following corrections may be carried out in the manuscript for its improvement,
Comment 4: Were the ions estimated in soil where the crop has been raised? And mention crop production methods, whether any nutrients were supplemented?
Author’s response: We did not measure ion content in the soil of the field where plants were grown, but, as far as we know, no ion deficiency/toxicity in the soil of the region nearby was reported. The field experiment was fertilized as per the usual farmer-based fertilization recommended in the region. In fact, the quantities of ions that we observed in the grains are within the order of magnitude of the ion contents usually observed in rice as mentioned in the article. Therefore, we do not suspect that a bias related to the abnormal presence of an ion in the soil has occurred.

Comment 5: Line 168, “Means, standard deviations, and coefficients of variation” may be modified as “Mean, standard deviation, and coefficients of variation”
Author’s response: We have edited the text accordingly.

Comment 6: Line 175, mention the model which has been used for calculation of BLUEs for your incomplete block design.
Author’s response: We have clarified the sentence indicating the R package used (StatgenSTA), the function within the package (SpATS) and the equation describing the model.

Results: The results has been presented well in different subsections viz., Variation in grain ion content in Vietnamese rice landraces, Identification of QTL related to grain ion content in Vietnamese rice landraces and Potential candidate genes related to grain ion content in Vietnamese rice landraces. However, the following corrections may be carried out in the manuscript for its improvement,
Comment 7: Line 220, “analyses were performed” may be modified as “analyses was performed”
Author’s response: We have edited the text accordingly.

Comment 8: Line 227, it is “(R < -0.34)” or “(R = -0.34)”? check all instances.
Author’s response: We have changed the text and now indicate the coefficient of correlation for both brown rice and white rice.

Comment 8: Line 228, it is “co-variations” or “correlations”?

Author’s response: We have now used the term “associations” to indicate that ion content in brown rice may be associated with ion content in white rice as illustrated by the correlations for P, K and Mg between the two grain types.

Comment 9: Table 2 may be modified for clarity. It should be organised based on the traits (ions). Additionally, points in the results section should reflect in the table (model in which it was associated, bonferroni threshold, etc.).
Author’s response: We have modified Table 2 according to the Reviewer’s comment.

Comment 10: Line 247-258, these results may also be added for investigation of candidate genes in Table 3. The relevance for these genes with associated traits should be established, or else delete it.
Author’s response: These sentences refer to colocalisation between the QTL associated with ion content found in this study and QTL associated with other traits in the same rice collection. None of these former QTL correspond to those in which we have identified interesting candidate genes. Therefore, the colocalization information cannot be added to Table 3. In Table 3 we have removed the LOC_Os04g42930 gene in qRMn4-1 as we acknowledge its function was not clearly associated with Mn acquisition and translocation.

Comment 11: Line 266, is it 3.6% or 3.6ppm? check all instances
Author’s response: We confirm that the T allele induces an increase in grain P content of 3.6% on average. We have changed the sentence to “3.6% increase in average” in qRP5-1. We have also changed the sentence describing the reduction of 24% in average for As content provided by the C allele in qRAs-10-1.

Comment 12: Line 261-287, the paragraphs looks more like a discussion. Modify it. The results section for these should only contain the findings of this study.
Author’s response: We appreciate the Reviewer’s feedback. However, we support the selection of these genes by their presence within the QTL interval which we determined, and their expression data or functional information which has been retrieved from databases or the references we are citing in this section. As such, this section presents information that we believe are appropriately placed and necessary within the results section, particularly as we were careful not to make this redundant with the discussion. This structure is also consistent with our previous publications (Debieu et al., 2018 and Affortit et al., 2022), and for these reasons, we have decided to retain these information in the results rather than moving it to the discussion.

Discussion: The discussion has been presented well and schematically organised. However, the following corrections may be carried out in the manuscript for its improvement,
Comment 13: Line 311, modify “with K, Mg, P showing smallest variation and Fe, Zn” as “with K, Mg and P showing smallest variation, while, Fe, Zn”
Author’s response: We have changed the text according to the Reviewers comment.

Abstract: The abstract is well drafted and sequentially written which reflects the contents of the manuscript. However, the corrections if made in the above sections need to be addressed in abstract also.
Author’s response: We have edited the abstract accordingly.

General comments:
1. Check whether the reference format is according to the journal style

Author’s response: We have revised the reference to align with the journal’s style.

2. Grammatical and typographic errors may be checked and corrected

Author’s response: We have diligently corrected these errors throughout the text. In addition, the paper underwent English editing by a private company.

**Reviewer #3:** Overall the findings of the study is compelling. Although there are few typographical error in some statements that could also be addressed, the efforts made by the authors are appreciable. Moreover, the findings of this study is almost agreed to support the previous works, QTL mapping studies for instance.

Author’s response: We thank the Reviewer for the constructive comments on the manuscript.

However, I have listed few corrections for the authors to look over it.
•Correct the typographical and grammatical errors in the following line numbers, [98,112,119,155 169](about:blank) etc…

Author’s response: We have diligently corrected these errors throughout the text. In addition, the paper underwent English editing by a private company.

•Did authors measured phytic acid levels? If so it would be good to included phytic acid contents in WR and BR grains

Author’s response: Indeed, it would have been interesting to measure phytic acid levels as it represents a strong chelator of metal cations and reduces the bioavailability of important micronutrients. However, due to budget limitations phytic acid were not measured in our experiments.

•Check for the references as per the journal format!

Author’s response: We have revised the reference to align with the journal’s style.
