# Supplementary material for: Quantitative trait loci for grain mineral element accumulation in Vietnamese rice landraces
Source: PLoS One. 2024 Dec 23;19(12):e0315666. doi: 10.1371/journal.pone.0315666 (PMC11666011; doi:10.1371/journal.pone.0315666)
Supplement: Attachment — Submitted filename: Response_To_Reviewers_Tran_etal_211124.docx [file pone.0315666.s010.docx]

**Reviewer #1:** It seems the authors have answered the queries raised by the reviewers in a more or less satisfactory manner and improvement in quality of MS in R1 version can be seen. However, the final call will be from editor's desk.

Author’s response: We thank the Reviewer for the comments on the manuscript.

Is the manuscript presented in an intelligible fashion and written in standard English? Reviewer #1: No

Author’s response: As stated in our previous round of revisions, the manuscript have undergone English language editing by the American Journal Experts company, and the editing certificate is included with the submission. We have further refined the clarity of the manuscript and made minor edits in the text. A tracked changes version is provided as part of the resubmission. We believe that these revisions have further improved the clarity of the manuscript. However, we remain open to addressing further clarification the reviewers may advise.

**Reviewer #2:** I have carefully reviewed the revised manuscript and the author’s responses to my comments. I am pleased to report that the authors have adequately addressed my suggestions and made the necessary revisions to improve the clarity and quality of the manuscript. I would suggest one more change to be made to improve the quality of the manuscript.

Author’s response: We thank the Reviewer for the comments on the manuscript.

Comment 1: Table 2, differentiate the SNPs that are associated in multiple GWAS models (14 SNPs) and which are exclusively associated in BLINK model (13 SNPs) using footnotes.

Author’s response: We have revised Table 2 in accordance with Reviewer 2’s suggestions.
